# Identification of major depressive disorder disease-related genes and functional pathways based on system dynamic changes of network connectivity

**DOI:** 10.1186/s12920-021-00908-z

**Published:** 2021-02-23

**Authors:** Ruijie Geng, Xiao Huang

**Affiliations:** 1 Department of Psychological Medicine, Zhongshan Hospital, Fudan University, Shanghai, 200032 China; 2 Department of Psychological Medicine, Xiamen Branch, Zhongshan Hospital, Fudan University, Xiamen, 361015 China

**Keywords:** Major depressive disorder, Anterior cingulate cortex, Prefrontal cortex, Co-expression network

## Abstract

**Background:**

Major depressive disorder (MDD) is a leading psychiatric disorder that involves complex abnormal biological functions and neural networks. This study aimed to compare the changes in the network connectivity of different brain tissues under different pathological conditions, analyzed the biological pathways and genes that are significantly related to disease progression, and further predicted the potential therapeutic drug targets.

**Methods:**

Expression of differentially expressed genes (DEGs) were analyzed with postmortem cingulate cortex (ACC) and prefrontal cortex (PFC) mRNA expression profile datasets downloaded from the Gene Expression Omnibus (GEO) database, including 76 MDD patients and 76 healthy subjects in ACC and 63 MDD patients and 63 healthy subjects in PFC. The co-expression network construction was based on system network analysis. The function of the genes was annotated by Kyoto Encyclopedia of Genes and Genomes (KEGG) pathway analysis. Human Protein Reference Database (HPRD, http://www.hprd.org/) was used for gene interaction relationship mapping.

**Results:**

We filtered 586 DEGs in ACC and 616 DEGs in PFC for further analysis. By constructing the co-expression network, we found that the gene connectivity was significantly reduced under disease conditions (*P* = 0.04 in PFC and *P* = 1.227e−09 in ACC). Crosstalk analysis showed that CD19, PTDSS2 and NDST2 were significantly differentially expressed in ACC and PFC of MDD patients. Among them, CD19 and PTDSS2 have been targeted by several drugs in the Drugbank database. KEGG pathway analysis demonstrated that the function of CD19 and PTDSS2 were enriched with the pathway of Glycerophospholipid metabolism and T cell receptor signaling pathway.

**Conclusion:**

Co-expression network and tissue comparing analysis can identify signaling pathways and cross talk genes related to MDD, which may provide novel insight for understanding the molecular mechanisms of MDD.

## Background

Major depressive disorder (MDD) is a leading psychiatric disorder, typically manifested as persistent depression, anhedonia, and occasional suicidal ideation and behavior [1]. The 12-month prevalence of this psychotic mood disorder is 10.4%, and the lifetime prevalence is 20.6% in United State [2]. MDD exerts negative effects on the quality of life and is also one of the leading causes of disability worldwide [3].

Although antidepressants are widely used at present, there are some limitations including long time treatment response (commonly weeks to months) and low response rates (one to two thirds will not respond to the first drug prescribed, and remain one third will not respond after multiple trials) [4–7]. The neuropathology mechanism underlying MDD remains unclear, which makes the diagnosis and treatment of depression be challenging.

In recent years, accumulating evidence suggests that depression is not only caused by a single brain region or a single gene abnormality but a disease with complex genetic characteristics and multiple etiologies. Widespread brain areas associated with “emotional network” were found to be abnormal in structure, function, and coordinated activity in MDD. Thus MDD is also considered as “disconnection syndrome”. Disturbances in brain activity and impaired mood regulation are considered to be the main neuropathology underlying depression [8]. Beyond the hippocampus, anterior cingulate cortex (ACC) and prefrontal cortex (PFC) are also common abnormal areas in MDD [9]. ACC is involved in the modulation of negative affect, pain and cognitive control [10]. PFC plays an important role in the regulation of the hypothalamo-pituitary-adrenal (HPA)-axis in stress response and also depression. There is increasing evidence that MDD and chronic stress are associated with an excitatory inhibition (E: I) imbalance within PFC which is caused by a deficit of inhibitory synaptic transmission onto principal glutamatergic neurons [11]. A recent fMRI study showed that the functional connectivity of the medial PFC in MDD patients is reduced [12]. Accordingly, depression is a heterogeneous syndrome with distinct causes and pathophysiology.

Gene expression analysis has found a large number of genes and disease-related information in MDD. But due to heterogeneity and various sources of noise, the discovery of pathogenesis is still limited [13–15]. Considering the fact that complex phenotypes manifested in mammalian systems are the result of a complex array of networks operating within and between tissues, a network perspective is necessary to explain its etiology. Tissue-to-tissue network analysis provides a method for the identification of disease-specific genes in response to abnormalities of tissues based on genome-wide association studies [16, 17]. Converging evidence indicated that gene co-expression studies offer complementary perspectives on gene changes in the context of transcriptome studies [18]. Co-expression genes possibly shared similar functions, and they may arise via multiple and diverse biological pathways such as common regulatory pathways [13, 16, 18]. Dysfunction of signaling pathways is likely to induce a variety of pathologies [19]. Notably, by integrating multiple interactions among a large number of genes, the study of gene co-expression networks provides an approach to tackle the complexity of biological changes in polygenic disease [13, 20].

In this study, we systematically integrated the postmortem brain (ACC and PFC) datasets of MDD patients and healthy subjects and constructed DEGs co-expression networks. We hypothesize that genes with correlated expression patterns across tissues are more likely to be related to the disease. This will provide a novel and powerful framework to improve the understanding of the molecular mechanisms of MDD.

## Methods

### Expression profile analysis

Datasets of mRNA expression profiles were downloaded from GEO database, including six postmortem ACC datasets ('E-GEOD-54572′, 'E-GEOD-54571′, 'E-GEOD-54565′, 'E-GEOD-54564′, 'E-GEOD-54563′, 'E-GEOD-54562′) [21] and six PFC datasets ('E-GEOD-54570′, 'E-GEOD-54568′, 'E-GEOD-54567′, 'E-GEOD-45642′, 'E-GEOD-35978′, 'E-GEOD-12654′) [14, 21–23]. Gene ID was converted into a gene symbol through the platform transformation. Multiple probes may correspond to one gene, therefore these probes were combined using the ‘WGCNA’ package in R platform[24]. To merge the expression profile data of multiple batches and platforms under the same variance level, we performed Z-test correction on all expression profile data. After integrating the data, two expression profile datasets of the ACC and PFC were obtained. The ‘limma’ R package was used to identify the DEGs (*P* < 0.05, |log2 (foldchange)|> 0) [25].

### Correlation analysis and Co-expression network construction

Compared with normal cellular homeostasis, gene expression pattern changes in disease conditions. The Changes in the correlation between genes can be used to identify critical genes related to the development of depression [26]. Therefore, we used the Pearson correlation coefficient with a threshold of 0.5 for correlation analysis to identify gene pairs that are significantly correlated between normal and disease states. Subsequently, we obtained gene co-expression relationships from the two brain tissues. The co-expression network was constructed by taken co-expression relationships as edges and genes as nodes. The isolated nodes and self-interactions were removed. Cytoscape software [27] (http://www.cytoscape.org) was applied for the construction of the network.

### Comparative analysis on difference of network

Generally speaking, gene interaction network follows power law distribution with stability and robustness. Compared with other genes, the hub genes in the network have a significantly higher number of connections. Gene connections in biological networks are dynamic and may lose or gain connections in the disturbed network under disease conditions [26]. Genes with altered connectivity or expression in disease are more likely to participate in disease progression and are expected to become therapeutic targets. Therefore, we statistically measured the gain or loss of nodes in the normal and disease networks.

### Functional pathway analysis

To further understand the biological functions of the DEGs from the two brain tissues, functional enrichment analysis was performed using KEGG (the Kyoto Encyclopedia of Genes and Genomes) pathway enrichment analysis (http://www.kegg.jp/). Enrichment methods used Fisher's exact test, the *P* values were adjusted by FDR (false discovery rate). Signaling pathways with *P* < 0.05 were considered as significantly enriched pathways.

### Cross talk analysis

Biological processes are interconnected and regulated by signal proteins. The pathways that influence the dynamics of each other are collectively called cross-talk [28]. Previous studies have found the possibility of crosstalk genes as important drug targets and biomarkers of diseases [29]. We used the DEGs and their enriched signaling pathways to construct a regulatory network. Human Protein Reference Database (HPRD, http://www.hprd.org/) was used for gene interaction relationship mapping. Based on the distribution of significant DEGs in each pathway, we identified crucial cross-talk genes that have a function in multiple important biological pathways.

## Results

### Expression profile analysis

**Fig. 1:**
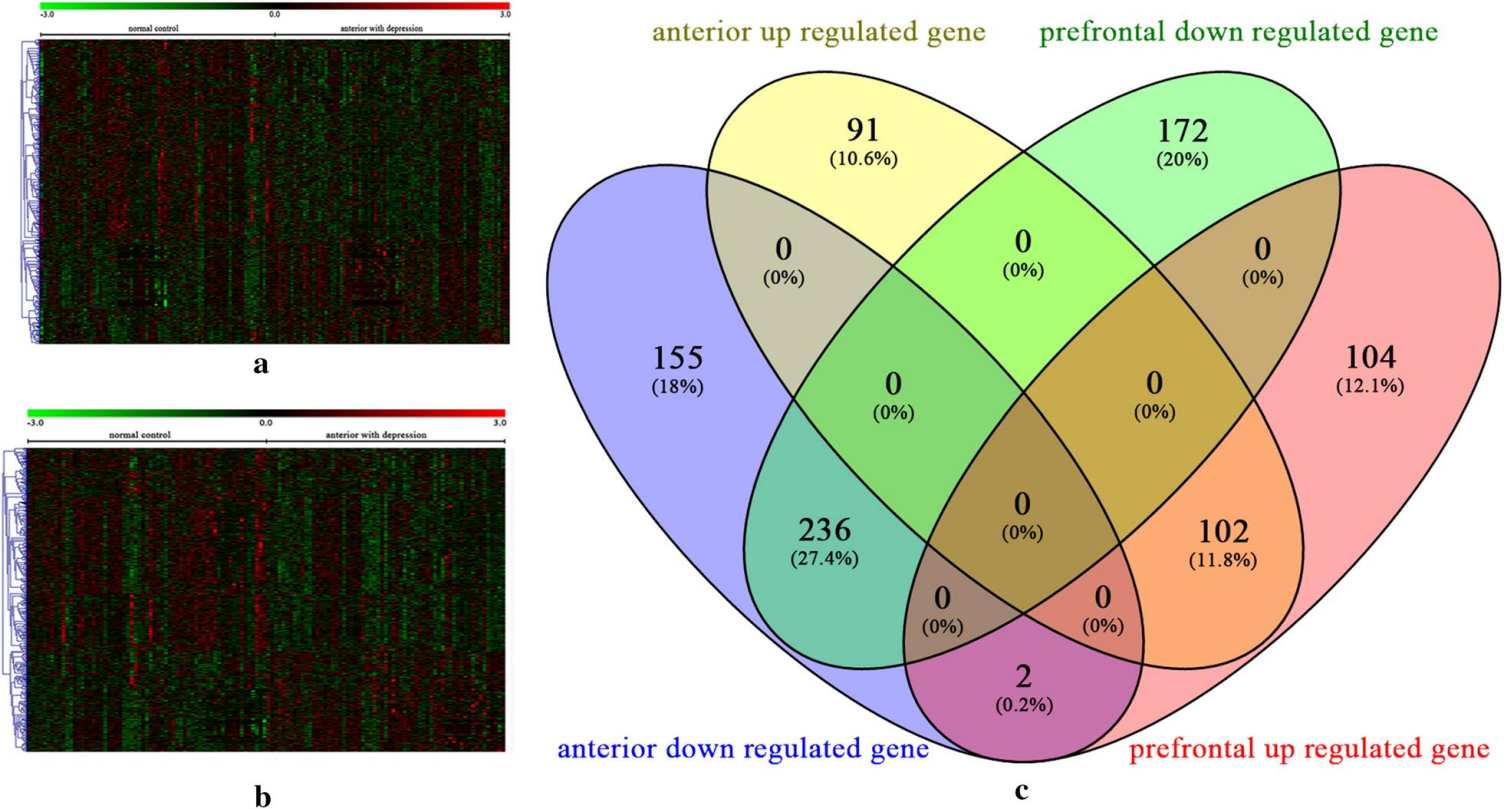
The differentially expressed genes (DEGs) in ACC and PFC of patients with depression. **a**, **b** Heatmap of DEGs in ACC and PFC, respectively. **c** The venn graph of DEGs between ACC and PFC. ACC, anterior cingulate cortex; PFC, prefrontal cortex

The integrated datasets of ACC contained 152 samples, including 76 disease samples and 76 healthy controls. While integrated datasets of PFC included 126 samples with 63 disease samples and 63 healthy controls. After differential gene analysis, 586 DEGs were obtained in ACC (Fig. 1a) and 616 DEGs in PFC (Fig. 1b). More than 50% of DEGs in the two tissues were dysregulated and most DEGs were down-regulated (Fig. 1c).

### Co-expression network construction

**Fig. 2:**
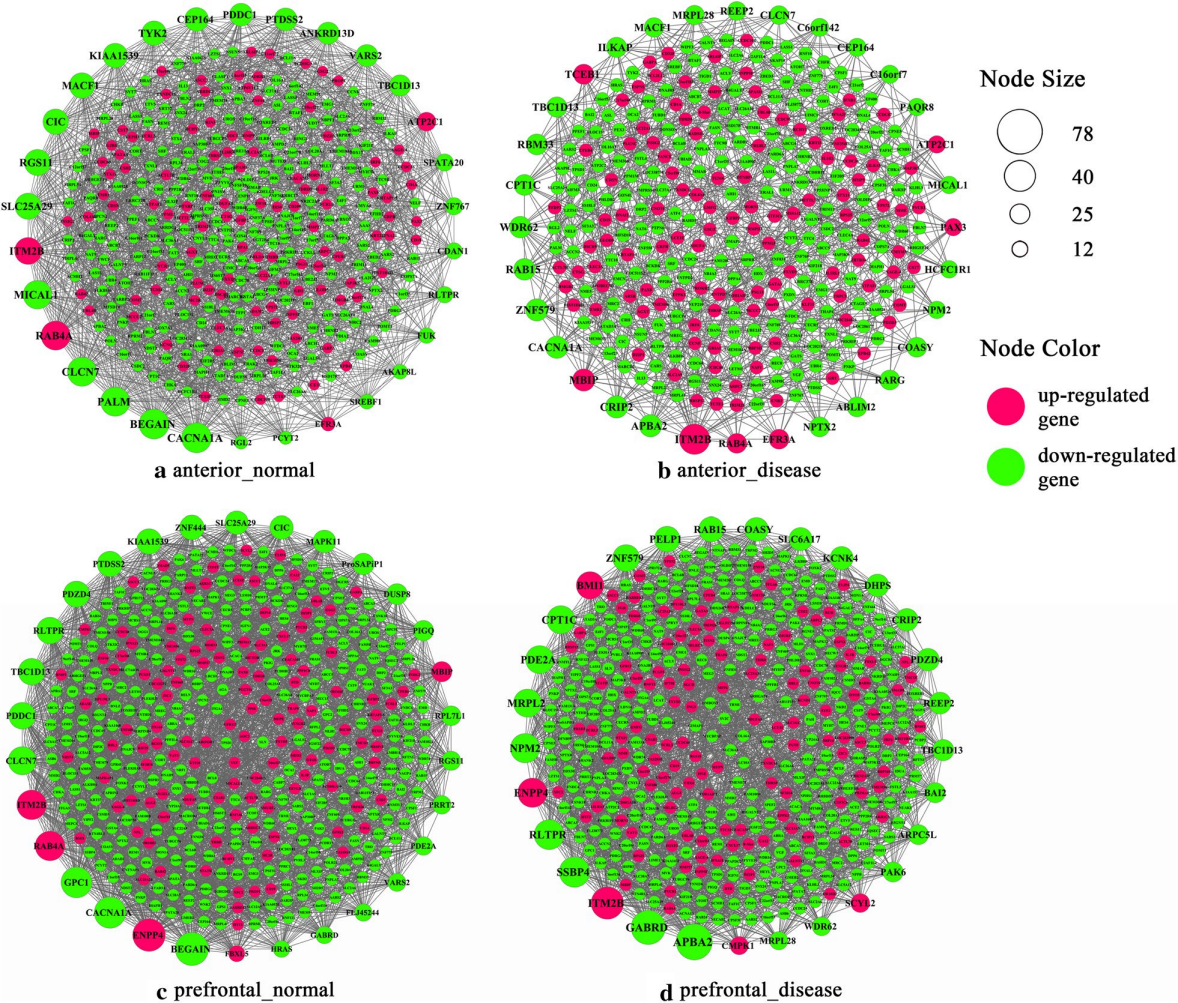
The co-expression network of **a** ACC normal, **b** ACC disease, **c** PFC normal and **d** PFC disease. ACC, anterior cingulate cortex; PFC, prefrontal cortex

We constructed co-expression networks based on the correlation between genes by Cytoscape software (Fig. 2) and analyzed the topological properties of these networks. Compared with normal conditions, there is no significant change in the number of nodes in the network under disease conditions, but the connectivity between genes is significantly reduced (Table 1). We used Wilcox test to calculate *p*-value for the difference in gene connectivity between normal and disease conditions. The results showed that *P* = 0.04 in PFC and *P* = 1.227e−09 in ACC. This suggested that under disease conditions, especially in ACC, there are statistically significant differences in the gain or loss of gene linkage.

**Table 1:** The comparison of network topological properties

summary	Anterior_normal	Anterior_disease	Prefrontal_normal	Prefrontal_disease
Nodes	388	346	465	472
Edges	1604	735	2941	2287
Unconnected nodes	89	111	68	92
Clustering coefficient	0.295	0.181	0.301	0.275
Density	0.021	0.012	0.027	0.021
centralization	0.101	0.078	0.141	0.118

### Comparative analysis on difference of networks

**Fig. 3:**
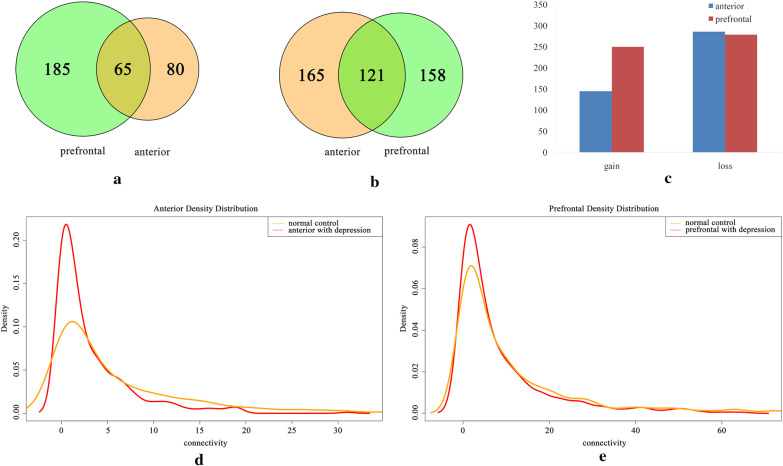
Comparative analysis of network difference. **a** The node counts of gain of connections between ACC and PFC; **b** The node counts of loss of connections between ACC and PFC; **c** Comparison of nodes with gain or loss of connections in ACC and PFC; **d** Probability density distribution of co-expression network in ACC under normal and depression conditions; **e** probability density distribution of co-expression network in PFC under normal and depression conditions. ACC, anterior cingulate cortex; PFC, prefrontal cortex

Network topology analysis reveals that gene connectivity was decreased in MDD patients. To further reveal the correlation of the two tissue lesions and MDD, we compared the nodes with gain and loss of connections (Fig. 3a, b) in the two disease networks. The number of nodes with a gain of connections was nearly balanced with the loss in PFC (Fig. 3c). Our data also showed that the number of nodes with a gain of connections in the PFC network was higher than that in the ACC network with the ratio of 1.72:1, while the numbers of nodes with loss of connections tended to be similar in the two networks with the ratio of 1.02:1. The probability density distribution of the co-expression network showed that the variance of the density distribution in ACC increases significantly (Fig. 3d), while it tends to be normal in PFC (Fig. 3e).

### Functional pathway analysis

**Fig. 4:**
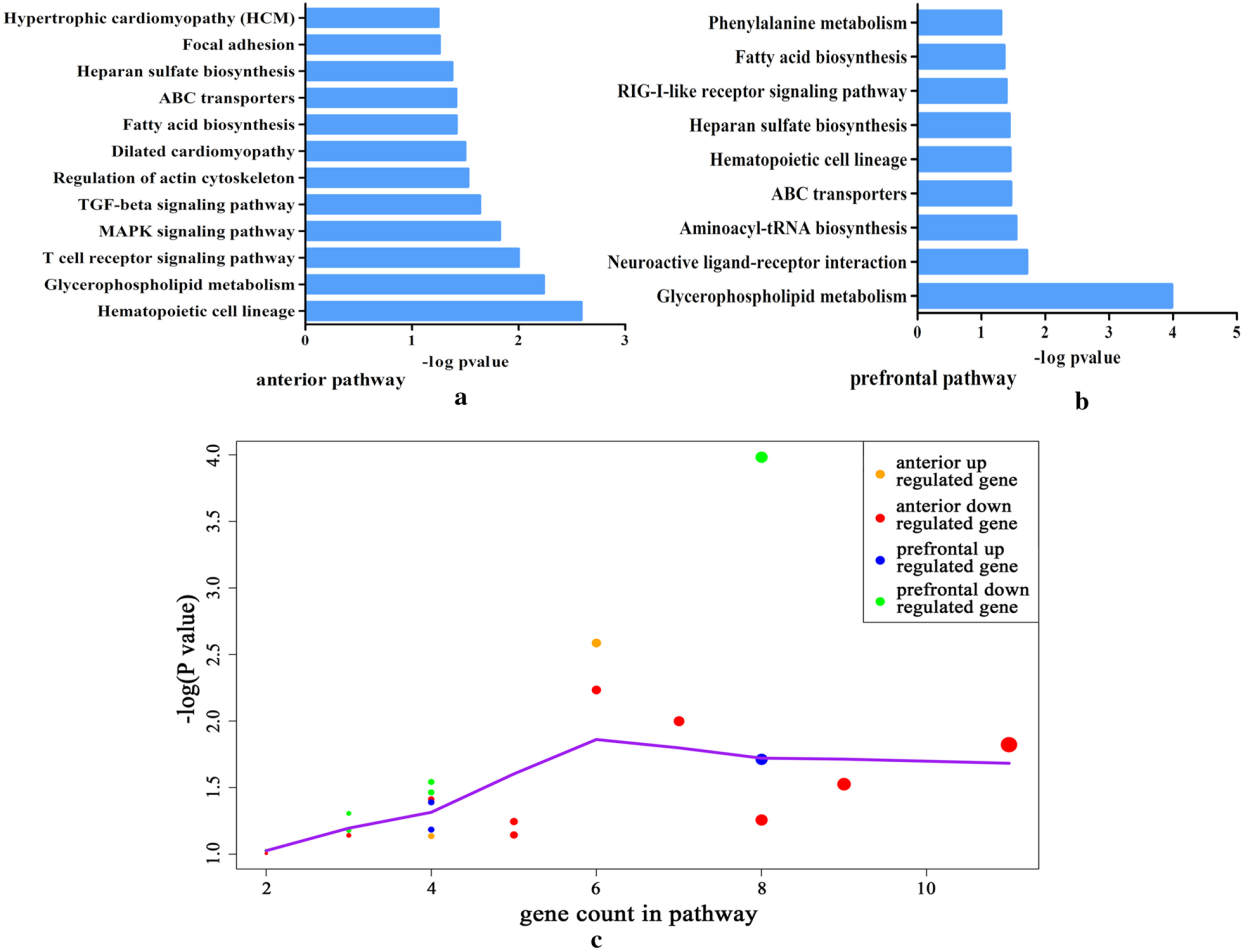
The pathway analysis for the DEGs of **a** ACC and **b** PFC. **c** Comparative analysis on gene count and pathway *p* value. The size of the nodes represents the number of DEGs that hit in the pathway; the bigger the size of node, the greater the count. The purple line represents linear fitting. ACC, anterior cingulate cortex; PFC, prefrontal cortex

To obtain information about the biological effects of DEGs, we performed the functional enrichment analysis on up-regulated and down-regulated DEGs. We found that DEGs in ACC were mainly associated with circulatory system related pathways (Fig. 4a), while DEGs in PFC were enriched in metabolic system related pathways (Fig. 4b). By comparing the gene count and the* P* value of the pathway, it is found that the P-value of the pathway increases with the increase of gene count (Fig. 4c). We further performed cross talk analysis on these DEGs enriched pathways, to identify the cross talk genes that regulate multiple signaling pathways.

### Cross talk analysis

**Fig. 5:**
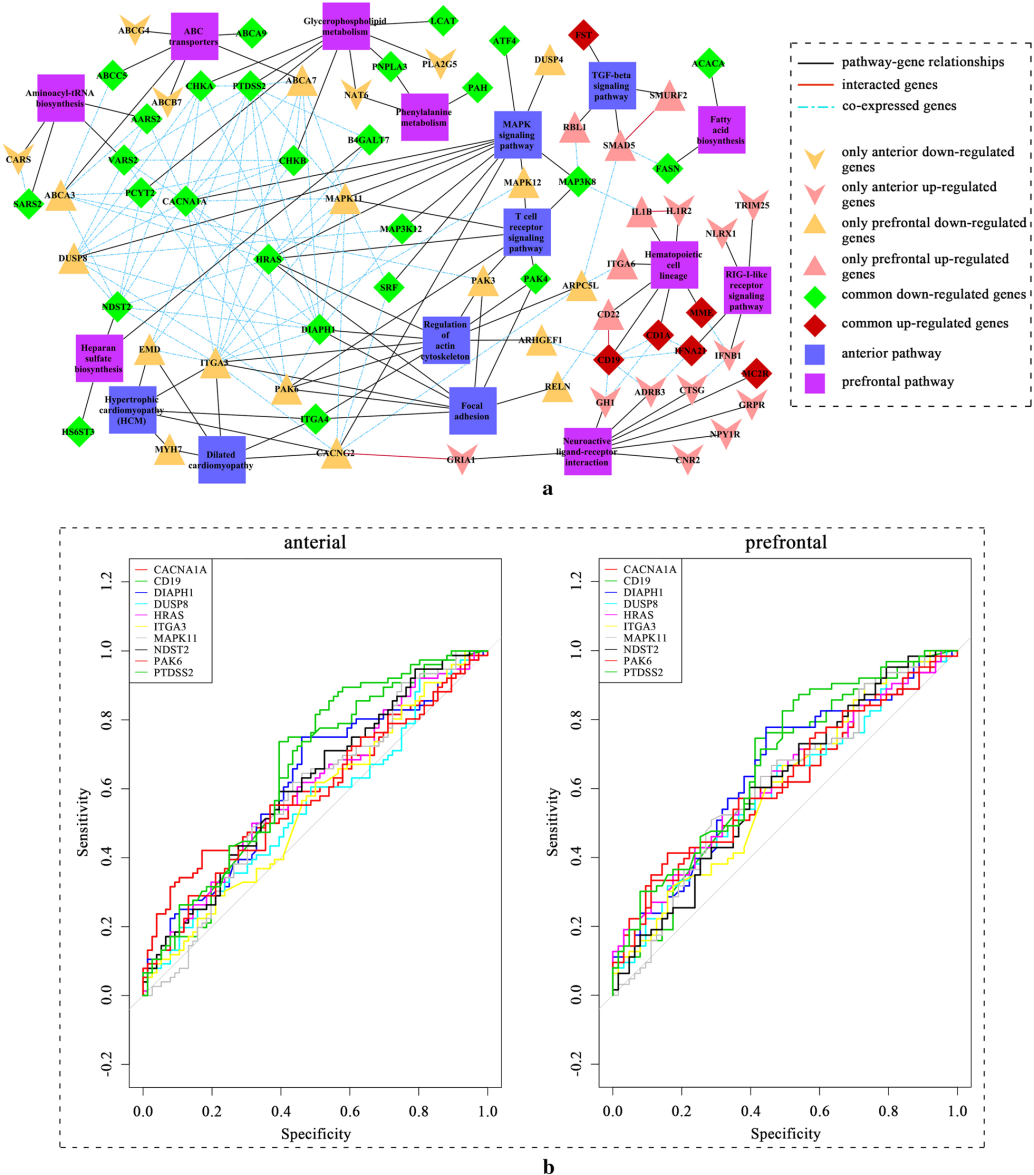
The pathway-gene complex network. **a** Pathway-gene complex network. **b** The ROC curve of top 10 genes in network

Signaling pathways and genes enriched in pathways were used to establish a pathway-gene complex network including 219 relationship pairs, 16 signaling pathways and 70 genes (Fig. 5a). The signaling pathways and genes with the highest degrees ranking as top 10 were extracted (Table 2). These top10 pathways and genes are more likely to be involved in the development of MDD and may also be potential new therapeutic targets.

**Table 2:** Top10 pathways and genes in the pathway-gene complex network

Pathway	Degree_path	Gene	Degree_gene
Glycerophospholipid metabolism	14	CACNA1A	22
MAPK signaling pathway	11	PTDSS2	19
Hematopoietic cell lineage	10	DIAPH1	15
Regulation of actin cytoskeleton	9	ITGA3	14
Focal adhesion	8	HRAS	14
Neuroactive ligand-receptor interaction	8	MAPK11	11
ABC transporters	8	DUSP8	11
T cell receptor signaling pathway	7	CD19	11
Heparan sulfate biosynthesis	6	PAK6	10
Dilated cardiomyopathy	5	NDST2	10

We further statistically analyzed the significance of the top10 genes through group comparison between ACC and PFC in MDD patients and healthy subjects (Table 3). Five genes (*CACNA1A, PTDSS2,* DIAPH1, *CD19 and* NDST2) in PFC and three genes (*PTDSS2, CD19 and* NDST2) in ACC were found differentially expressed in MDD patients (Table 3). Receiver operating characteristic (ROC) values of genes were calculated in the two tissues. All the ROC values of genes were higher than random state (0.5) (Fig. 5b and Table 3). To examine whether these top10 genes are capable to be potential novel therapeutic targets, we searched these genes in the Drugbank database. As shown in Table 4, five genes (*CACNA1A, PTDSS2, MAPK11*, *CD19 and PAK6*) were known to be targeted by several drugs. Except for Blinatumomab and KC706, other drugs have been reported to be related to brain tissue injury and cerebral nervous system diseases.

**Table 3:** Significant analysis using top 10 genes

Gene	*P* value_anterior vs control	*p*_*corrected*_ anterior vs prefrontal	*P* value_prefrontal vs control	*p*_*corrected*_ prefrontal vs control	ROC_anterior	ROC_prefrontal
CACNA1A	0.031	0.062	0.031	0.044	0.606	0.615
PTDSS2	0.003	0.013	0.009	0.030	0.641	0.643
DIAPH1	0.016	0.054	0.008	0.039	0.616	0.644
ITGA3	0.270	0.270	0.072	0.270	0.550	0.600
HRAS	0.032	0.054	0.022	0.054	0.598	0.620
MAPK11	0.256	0.285	0.046	0.051	0.549	0.600
DUSP8	0.086	0.122	0.030	0.050	0.583	0.610
CD19	0.017	0.044	0.035	0.044	0.612	0.608
PAK6	0.128	0.160	0.030	0.060	0.576	0.619
NDST2	0.0007	0.008	0.0009	0.009	0.663	0.680

**Table 4:** Drugable target information for the top 10 genes

Gene	Target (yes/no)	Drug counts	Drugs	PMID
*CACNA1A*	Yes	4	Amlodipine, loperamide, lyrica, pregabalin	25918454, 26390138, 26138193, 26670374
*CD19*	Yes	1	Blinatumomab	
*PAK6*	Yes	4	Dextromethorphan, Tizanidine, Agmatine, Moxonidine	26471212, 23648652, 26678503, 24333661
*MAPK11*	Yes	2	KC706, Regorafenib	25563977
*PTDSS2*	Yes	1	Phosphatidylserine	26689775
DIAPH1	No	0		
ITGA3	No	0		
HRAS	No	0		
DUSP8	No	0		
NDST2	No	0		

## Discussion

In the current study, we analyzed DEGs in ACC and PFC from patients with MDD. Correlation networks based on co-expression were constructed. Topological properties of the networks were analyzed and compared. Our results showed that the lesions of brain tissues in MDD patients were not synchronized and alterations of biological functions were not consistent either. ACC showed a greater degree of abnormality as compared to PFC suggesting a higher correlation with disease progression. We consequently analyzed the signaling pathways enriched by DEGs and further cross talk genes that bridge the multiple pathways were also identified. Through the construction of the pathway-gene complex network, the genes and singling pathways with top10 degrees were extracted, which are more likely to be potential novel therapeutic targets. We also mined the drugbank database for the top10 cross talk genes to explore their drugable target potential. *PTDSS2* and *CD19* differentially expressed in both ACC and PFC may correlate with MDD progression, and are more likely to become the new drug targets for the treatment of MDD.

The co-expression network has the ability to mine functionally related genes with similar co-expression patterns [30], which have been widely used to identify candidate biomarkers and therapeutic targets for many complex diseases, such as Alzheimer’s disease, schizophrenia and cancer [31–33]. Besides, the network perspective supports the high heterogeneity of depression and explains how different treatment methods might take effect [8, 20]. Comparisons between many data sets can provide a global view of gene expression patterns across tissues [16]. Therefore, we completed a comprehensive analysis of gene expressions across ACC and PFC in patients with MDD and healthy subjects. Through analyzing and comparing the four co-expression networks, we found the unconnected nodes were increased in disease condition, which may be due to loss of connections. Alteration in important nodes of the network may affect the function of the entire network, causing depression. The topology analysis of the co-expression network showed that in disease states, the number of nodes with a gain of connections in PFC network higher than that in ACC network with the ratio of 1.72:1 and variance of density distribution was markedly increased in the ACC network, but there is no significant change in PFC. These results indicated that the PFC network status of patients with depression tended to be normal, while the ACC network presented drastic fluctuations. The stability of the PFC network and its resistance to disease signals are better than that of the ACC network, which was consistent with the results of the Wilcox test. It revealed that the pathological changes of brain tissues in depression patients were not synchronized and alterations of biological functions were not consistent either. Compared with PFC, ACC showed a higher degree of abnormality and may have a strong correlation with disease progression. Therefore, ACC is more likely to be a therapeutic target for depression. ACC is located in the frontal part of the cingulate cortex inside the cerebral hemispheres and is a part of the limbic system. Substantial evidence from healthy subjects has linked the ACC to emotional behavior [10, 34]. This brain area uses information about punishment to manage aversively motivated actions. Bush et al. compiled a large amount of functional imaging, electrophysiological and anatomical data, and found that the ACC is specialized for affective processes [35]. Philippi et al. used resting-state fMRI to examine the functional connectivity of the ACC subregion in 28 participants with subclinical levels of depression. The results suggested that there is a clear correlation between depression severity and functional connectivity of ACC subregions. The reduced pregenual ACC-striatum connectivity and anterior subgenual ACC -anterior insula connectivity was related to higher depression severity [36]. Similarly, our research also found that ACC network connectivity in patients with depression has decreased, which is consistent with previous studies.

Signaling molecules commonly do not work individually but interact with other proteins or biological molecules to achieve signal transmission. for gaining further understanding of MDD. Moreover, these signaling components which may co-expressed in a dataset and correlate across samples are predicted to reconstruct multiple signaling pathways and their cross-talk maps for further biomedical research [16]. Cross-talk analysis is commonly used to explore the regulation and cooperation between signaling pathways, and further reveal the pathogenesis of diseases [37]. Therefore, through analyzing the pathway-gene network, we identified ten pathways and ten cross talk genes with highest degrees. Among these 10 genes, *CD19*, *PTDSS2* and *NDST2* were significantly differentially expressed in ACC and PFC of MDD patients. Moreover, *CD19* and *PTDSS2* have been targeted by several drugs. Therefore, these two genes may be related to the progression of MDD or other neurological diseases, and are more likely to become the new drug targets for the treatment of MDD.

Phosphatidylserine synthase 2 (*PTDSS2*) can convert phosphatidylethanolamine (PE) into phosphatidylserine(PS) and participate in important cell signaling processes [38, 39]. Compared with other tissues, brain is enriched in PS and PE. Besides, > 36% of the PS are composed of docosahexaenoic acid (DHA) which is essential for normal function of the nervous system [40, 41]. Studies have shown that the reduction of DHA is associated with the development of mild cognitive impairment to Alzheimer's disease [42]. Similarly, we observed that the most significant pathway is Glycerophospholipid metabolism. Both this pathway and PTDSS2 are related to lipid metabolism. Recent studies have shown that meningeal lipids play an important role in the pathogenesis of depressive disorder and anxiety [39]. The typical glycerophospholipids (GPLs) found in mammalian membranes include phosphatidylcholines (PC), PE, PS and phosphatidylinositols (PI) that are all attached through a phosphodiester linkage [39]. Preclinical findings indicated that the membrane-forming n-3 polyunsaturated fatty acids, glycerolipids, GPLs, and sphingolipids (SPLs) play a crucial role in the induction of depression- and anxiety-related behaviors [43]. Clinical studies suggested that compared with non-depressed non-suicide subjects, the activities of phosphatidylinositol 3-kinase (PI3K) and Akt (serine threonine kinase or protein kinase B) in MDD patients were significantly reduced [44]. Another crucial gene, *CD19*, is a B cell-specific member of the immunoglobulin superfamily expressed by pre-B cells from the time of heavy chain rearrangement to final differentiation into plasma cells. By regulating B cell receptor signaling, *CD19* guides the fate of B cells and differentiation lymphopoiesis [45]. In our study, *CD19* is the top gene in the T cell receptor signaling pathway that participates in immune regulation and inflammatory response. Immunity dysfunction is a risk factor for depression. Large clinical cohort studies have found that autoimmune diseases or severe infections increase the risk of mood disorders [46]. The activation of innate immune cells produces pro-inflammatory cytokines, which can cause major depressive disorder by inhibiting monoamine neurotransmitters, activating the HPA axis, and affecting neurogenesis and plasticity [47]. From the clinical perspective, anti-inflammatory drugs, such as minocycline, have been reported to cause improvement in patients with treatment-resistant depression [48]. Taken together, improving lipid metabolism and regulating inflammatory response can provide new directions for the prevention and treatment of MDD.

Our research has identified several critical genes and provided some interesting clues for further experiments. However, some limitations of the study should be mentioned. First, we identified several genes from microarray data analysis. But we did not perform further functional verification of these selected genes. Subsequently, a large number of clinical samples will be needed to validate our findings and clarify the underlying mechanisms of how these genes affect the pathological stage. Another limitation of the study is the AUC of the curve is low, although the ROC values of all genes are higher than the random state (0.5). Therefore, the interpretation of this result needs to be cautious. Further exploration is needed in the future.

## Conclusions

In conclusion, this study can identify several crucial genes for future genetic association studies. It also proved the essence of integrating cross-tissue data, gene co-expression and crosstalk signaling results, paving the way for novel and complementary approaches to investigate the molecular pathology of MDD and other complex brain disorders.

## Data Availability

The GeneChip data were retrieved from the GEO data repository (http://www.ncbi.nlm.nih.gov/geo/) with the accession numbers GSE54572, GSE54571, GSE54565, GSE54564, GSE54563, GSE54562, GSE54570, GSE54568, GSE54567, GSE45642, GSE35978, GSE12654.

## Abbreviations

MDD: Major depressive disorder
ACC: Postmortem anterior cingulate cortex
PFC: Prefrontal cortex
DEGs: Differentially expressed genes
KEGG: The Kyoto Encyclopedia of Genes and Genomes)
FDR: False discovery rate
HPRD: Human Protein Reference Database
ROC: Receiver operating characteristic
BDNF: Brain-derived neurotrophic factor
GABA: Gamma amino acid butyric acid
GPLs: Glycerophospholipids
PC: Phosphatidylcholines
PE: Phosphatidylethanolamines
PS: Phosphatidylserines
PI: Phosphatidylinositols
PI3K: Phosphatidylinositol-3 kinase
SPLs: Sphingolipids
mTORC1: MTOR complex 1
PSD95: Postsynaptic density-95
PTDSS2: Phosphatidylserine synthase 2
PAKs: P21-activated kinases
AR: Androgen receptor
GR: Glucocorticoid receptor
